# Spontaneous reperfusion enhances succinate concentration in peripheral blood from stemi patients but its levels does not correlate with myocardial infarct size or area at risk

**DOI:** 10.1038/s41598-023-34196-7

**Published:** 2023-04-27

**Authors:** Marta Consegal, Ignasi Barba, Bruno García del Blanco, Imanol Otaegui, José F. Rodríguez-Palomares, Gerard Martí, Bernat Serra, Neus Bellera, Manuel Ojeda-Ramos, Filipa Valente, Maria Ángeles Carmona, Elisabet Miró-Casas, Antonia Sambola, Rosa María Lidón, Jordi Bañeras, José Antonio Barrabés, Cristina Rodríguez, Begoña Benito, Marisol Ruiz-Meana, Javier Inserte, Ignacio Ferreira-González, Antonio Rodríguez-Sinovas

**Affiliations:** 1 Cardiovascular Diseases Research Group, Department of Cardiology, Vall d’Hebron Institut de Recerca (VHIR), Vall d’Hebron Hospital Universitari, Vall d’Hebron Barcelona Hospital Campus, Passeig Vall d’Hebron 119-129, 08035 Barcelona, Spain; 2 Departament de Medicina, Universitat Autònoma de Barcelona, 08193 Bellaterra, Spain; 3 Centro de Investigación Biomédica en Red (CIBER) de Enfermedades Cardiovasculares (CIBERCV), Instituto de Salud Carlos III, Madrid, Spain; 4 Faculty of Medicine, University of Vic - Central University of Catalonia (UVicUCC), Can Baumann. Ctra. de Roda, 70, 08500 Vic, Spain; 5 Institut de Recerca Hospital de la Santa Creu i Sant Pau (IRHSCSP), Barcelona, Spain; 6 Centro de Investigación Biomédica en Red (CIBER) de Epidemiología y Salud Pública, CIBERESP, Instituto de Salud Carlos III, Madrid, Spain

**Keywords:** Cardiovascular biology, Diagnostic markers, Prognostic markers

## Abstract

Succinate is enhanced during initial reperfusion in blood from the coronary sinus in ST-segment elevation myocardial infarction (STEMI) patients and in pigs submitted to transient coronary occlusion. Succinate levels might have a prognostic value, as they may correlate with edema volume or myocardial infarct size. However, blood from the coronary sinus is not routinely obtained in the CathLab. As succinate might be also increased in peripheral blood, we aimed to investigate whether peripheral plasma concentrations of succinate and other metabolites obtained during coronary revascularization correlate with edema volume or infarct size in STEMI patients. Plasma samples were obtained from peripheral blood within the first 10 min of revascularization in 102 STEMI patients included in the COMBAT-MI trial (initial TIMI 1) and from 9 additional patients with restituted coronary blood flow (TIMI 2). Metabolite concentrations were analyzed by ^1^H-NMR. Succinate concentration averaged 0.069 ± 0.0073 mmol/L in patients with TIMI flow ≤ 1 and was significantly increased in those with TIMI 2 at admission (0.141 ± 0.058 mmol/L, *p* < 0.05). However, regression analysis did not detect any significant correlation between most metabolite concentrations and infarct size, extent of edema or other cardiac magnetic resonance (CMR) variables. In conclusion, spontaneous reperfusion in TIMI 2 patients associates with enhanced succinate levels in peripheral blood, suggesting that succinate release increases overtime following reperfusion. However, early plasma levels of succinate and other metabolites obtained from peripheral blood does not correlate with the degree of irreversible injury or area at risk in STEMI patients, and cannot be considered as predictors of CMR variables.

**Trial registration:** Registered at www.clinicaltrials.gov (NCT02404376) on 31/03/2015. EudraCT number: 2015-001000-58.

## Introduction

Previous studies demonstrated that succinate, the endogenous substrate used by the mitochondrial enzyme succinate dehydrogenase (or mitochondrial complex II), accumulates in ischemic tissues, including the myocardium^1–4^, and is massively released into the bloodstream after flow restoration^5–8^. Moreover, it was shown that succinate can be detected in plasma from STEMI patients immediately after stent implantation, both in blood obtained from a peripheral vein or from the coronary sinus, and that its plasma concentrations at the coronary sinus correlate with edema volume^5^. Interestingly, we have recently demonstrated that plasma levels of succinate and other citric acid intermediates are enhanced in the blood obtained from the great cardiac vein 5 min after reperfusion in a pig model of transient coronary occlusion, and that their concentration is reduced by protective maneuvers and correlates with final infarct size^8^.

However, obtaining blood from the coronary sinus is not included in the routine practice, is not always feasible and is not without risk. As most STEMI patients have a peripheral via, and as succinate concentration is also enhanced in blood from this origin^5^, we aimed to assess whether the concentration of succinate and other metabolites in peripheral plasma, obtained during the coronary procedure, correlates with myocardial edema or infarct size, as determined by CMR, in patients undergoing emergency primary percutaneous coronary intervention (PPCI) in our center (Vall d’Hebron Hospital Universitari, Barcelona, Spain) and included in the randomized COMBAT-MI clinical trial^9^.

## Methods

The present sub-study analyzes the plasma concentration of succinate and other metabolites in a subgroup of STEMI patients submitted to PPCI (n = 111) and included in the COMBAT-MI clinical trial (registered at www.clinicaltrials.gov (NCT02404376) on 31/03/2015; EudraCT number 2015-001000-58)^9^. The COMBAT-MI trial was a prospective, randomized, multicentric, double blinded, clinical trial comparing the effects of sham procedure, intravenous exenatide, remote ischemic conditioning (RIC), and their combination on infarct size measured by late gadolinium enhancement in CMR in patients with STEMI undergoing PPCI (allocation ratio 1:1:1:1 via a web-based clinical support system accessible 24 h a day (W3NEXUS, Barcelona, Spain); further details in^9^), and main results were published in^9^. The study was conducted in accordance with the Declaration of Helsinki and the European Guidelines for Good Clinical Practice, and was approved by the Agencia Española de Medicamentos y Productos Sanitarios (AEMPS) and the Ethics Committee of participant institutions.

Patients with diagnosis of STEMI, older than 18 years, presenting within 6 h of symptom onset were included in the original study^9^. STEMI was characterized by ischemic symptoms, including chest pain, and ≥ 1 mm ST elevation in 2 leads in the same territory or ≥ 2 mm ST elevation in ≥ 2 V1 through V4 leads or left bundle branch block with ≥ 1 mm concordant ST elevation. Exclusion criteria included TIMI flow grade at admission equal to 2 or 3. Additional exclusion criteria can be found in^9^. Patients eligible were recruited in our center between March 2016 and June 2019 and enrolled in the emergency room or upon entering the catheterization laboratory. Primary and secondary study endpoints can be found in^9^.

Although in the original study only patients with TIMI flow ≤ 1 were included, plasma samples were available for 102 patients who met the inclusion criteria and for 9 with TIMI flow equal to 2. PPCI followed guideline recommendations and was performed by experienced operators without any delay. Blood samples were obtained at the time of PPCI from the 111 STEMI patients randomized in our center (in 102 within the first 10 min after revascularization and in 9 with initial TIMI flow ≥ 2 during coronariography) and placed in EDTA tubes. Plasma was obtained after centrifugation at 1500 g for 10 min. The supernatant was then centrifuged again at 2500 g (15 min) and maintained at − 80 °C until use. CMR data were obtained 3–7 days after PPCI^9^.

### Analysis of plasma samples by nuclear magnetic resonance spectroscopy

Plasma metabolites were extracted using the methanol method and ^1^H-NMR spectra were acquired on a vertical bore 9.4 T magnet interfaced to a Bruker Avance 400 spectrometer, as previously described^10^.

### Statistics

Sample size calculation in the original COMBAT-MI clinical trial can be found in^9^. Data in this substudy are expressed as mean ± SEM. Differences in baseline characteristics and outcomes between patients with initial TIMI flow ≤ 1 and those with TIMI flow 2 were analyzed by Student’s t test. Linear regression analysis was used to assess the existence of correlations between metabolite concentrations and CMR variables. Predictors for myocardial infarct size, myocardial salvage index, transmurality index, left ventricular ejection fraction and microvascular obstruction volume, measured by CMR imaging, were determined by stepwise regression analysis. Differences were considered significant when *p* < 0.05.

### Ethics approval and consent to particate

The study was conducted in accordance with the Declaration of Helsinki and the European Guidelines for Good Clinical Practice, and was approved by the Agencia Española de Medicamentos y Productos Sanitarios (AEMPS) and the Ethics Committee of participant institutions. All patients provided written informed consent before randomization.

## Results

The numbers of participants who were randomly assigned, received intended treatment, and were analyzed for the primary outcome, together with losses and exclusions after randomization, can be found in^9^. Exploratory clinical adverse events during hospitalization can also be found in^9^.

For the present substudy, baseline clinical and procedural characteristics and CMR outcomes of the 102 patients originally included in the COMBAT-MI clinical trial (TIMI flow ≤ 1) were similar to those of patients with TIMI flow ≥ 2 (Table 1). The mean age of the pooled population was 61.41 ± 1.07 years, and 96 (85.5%) of the patients were male.

**Table 1:** Baseline clinical and procedural characteristics, comorbidities and CMR outcomes of patients with initial TIMI flow ≤ 1 versus those with TIMI flow at admission equal to 2.

		TIMI ≤ 1 (n = 102)	TIMI = 2 (n = 9)	*p* value
Male, nº (%)		87 (85.29%)	9 (100%)	NS
Age (years)		61.80 ± 1.15	56.89 ± 2.09	NS
Body weight (kg)		78.81 ± 1.22	83.89 ± 4.08	NS
Height (cm)		168.98 ± 0.78	174.33 ± 2.62	NS
BMI (kg/m^2^)		27.54 ± 0.35	27.77 ± 1.71	NS
Body surface area (BSA) (m^2^)		1.92 ± 0.02	2.01 ± 0.05	NS
Comorbidities:				
Smoking, nº (%)	Active	44 (43.14)	6 (66.67)	NS
	Ex-smoker	34 (33.33)	1 (11.11)	
Hypertension, nº (%)		44 (43.14)	5 (55.55)	NS
Dyslipidemia, nº (%)		59 (55.84)	6 (66.67)	NS
Diabetes, nº (%)	With diet	5 (4.90)	0 (0.00)	NS
	Insulin	4 (3.92)	0 (0.00)	
	OAD	17 (16.67)	2 (22.22)	
	Insulin + OAD	1 (0.98)	0 (0.00)	
Killip class	1	83 (81.37)	8 (88.89)	NS
	2	11 (10.78)	1 (11.10)	
	3	1 (9.80)	0 (0.00)	
	4	4 (3.92)	0 (0.00)	
Procedural details				
Infarct-related artery	RCA	48 (47.06)	4 (44.44)	NS
	LDA	44 (43.14)	4 (44.44)	
	LCX	10 (9.80)	1 (11.11)	
Symptom-to-door (min)		151.29 ± 7.09	134.44 ± 13.92	NS
Symptom-to-ballon (min)		168.25 ± 7.01	153.33 ± 14.29	NS
Sum of ST-seg. elevation		3.93 ± 0.33	4.71 ± 0.92	NS
Systolic pres. (mmHg)		131.91 ± 2.58	136.22 ± 5.09	NS
Diastolic pres. (mmHg)		78.28 ± 1.60	89.00 ± 4.92	NS
Heart rate (beats/min)		72.13 ± 1.53	83.67 ± 4.21	NS
TIMI flow (post-proced.)	TIMI = 1, nº(%)	1 (0.98)	0 (0.00)	NS
	TIMI = 3, nº(%)	7 (6.86)	2 (22.22)	
	TIMI = 4, nº(%)	93 (91.18)	7 (77.78)	
CMR outcomes				
Infarct size (% of LV mass)		23.60 ± 1.14	22.99 ± 3.48	NS
Infarct size (g)		29.88 ± 1.62	30.09 ± 4.95	NS
Myocardial salvage index (%)		9.09 ± .073	7.46 ± 1.46	NS
Transmurality index		47.70 ± 1.21	44.93 ± 3.47	NS
Heart rate (beats/min)		65.83 ± 1.17	72.11 ± 7.49	NS
LVEF (%)		45.00 ± 0.99	42.89 ± 3.90	NS
Extent of edema (g)		38.56 ± 1.85	37.56 ± 4.86	NS
MVO (%)		0.82 ± 0.16	0.81 ± 0.60	NS
Cardiac mass (g)		124.79 ± 2.79	129.48 ± 6.11	NS
Cardiac mass/BSA		64.59 ± 1.28	64.56 ± 2.93	NS

BMI, body mass index; BSA, body surface area; LCX, left circumflex coronary artery; LDA, left descending coronary artery; LV, left ventricular; LVEF, left ventricular ejection fraction; MVO, relative microvascular obstruction; OAD, oral antidiabetic drugs; pres., pressure; post-proced., post-procedure; RCA, right coronary artery; seg., segment.

### Metabolomic profile of plasma samples from STEMI patients by nuclear magnetic resonance spectroscopy

**Figure 1:**
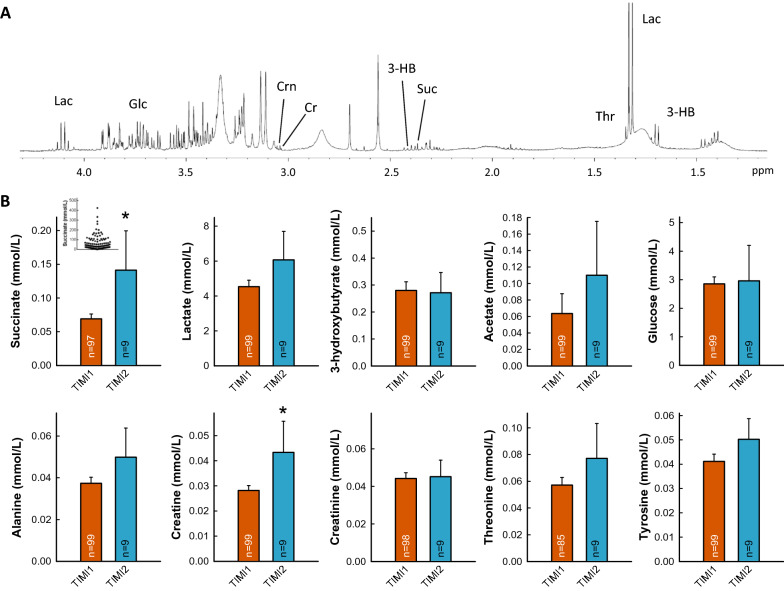
(**A**) Representative ^1^H-NMR spectra obtained from plasma extracts in a STEMI patient included in the COMBAT-MI clinical trial. Abbreviations: 3-HB: 3-hydroxybutyrate; Cr: Creatine; Crn: Creatinine; Glc: Glucose; Lac: Lactate; Suc: Succinate; Thr: Threonine. (**B**) Concentrations of selected metabolites (mmol/L) analyzed by ^1^H NMR spectroscopy in plasma extracts obtained from peripheral blood samples in STEMI patients with initial TIMI flow 1 as compared with data from those with initial TIMI flow 2. * (*p* < 0.05) indicates significant differences vs. TIMI 1 group. Inset shows variability in succinate concentrations in control TIMI 1 patients (data in µmol/L).

^1^H-NMR spectra of plasma extracts allowed identification of succinate and other metabolites including lactate, 3-hydroxybutirate, acetate, glucose, alanine, creatine, creatinine, threonine, and tyrosine. Succinate concentration averaged 0.069 ± 0.0073 mmol/L in patients with TIMI flow ≤ 1 (Fig. 1), in the range of previous studies^11^, and was significantly enhanced in the 9 additional patients with TIMI flow ≥ 2 (0.141 ± 0.058 mmol/L, *p* < 0.05, Fig. 1, Table 2). Similar trends were observed for other metabolites, especially creatine (Fig. 1).

**Table 2:** Concentrations of selected metabolites (mmol/L) analyzed by ^1^H NMR spectroscopy in plasma extracts obtained from peripheral blood samples in STEMI patients with initial TIMI flow 1 as compared with data from those with initial TIMI flow 2. * (*p* < 0.05) indicates significant differences versus TIMI 1 group.

	TIMI 1	TIMI 2
Succinate	0.069 ± 0.007	0.141 ± 0.058 *
Lactate	4.537 ± 0.369	6.071 ± 1.631
3-hydroxybutyrate	0.280 ± 0.032	0.272 ± 0.075
Acetate	0.064 ± 0.024	0.110 ± 0.065
Glucose	2.855 ± 0.242	2.962 ± 1.240
Alanine	0.037 ± 0.003	0.050 ± 0.014
Creatine	0.028 ± 0.002	0.043 ± 0.012 *
Creatinine	0.044 ± 0.003	0.045 ± 0.089
Threonine	0.057 ± 0.006	0.077 ± 0.026
Tyrosine	0.041 ± 0.003	0.050 ± 0.009

### Correlations between metabolite concentrations in peripheral plasma and CMR variables

Regression analysis did not show any significant correlation between the concentrations of the different metabolites analyzed, including succinate, and infarct size (determined as percentage of left ventricular mass or as absolute weight), myocardial salvage index, transmurality index, extent of edema, left ventricular ejection fraction (LVEF) or microvascular obstruction volume. Furthermore, with the exception of creatine for myocardial salvage index (*p* = 0.008) and transmurality index (*p* = 0.039), stepwise regression analysis did not identify any metabolite as predictor of any of the analyzed variables.

## Discussion

This study shows that the presence of restituted blood flow at the culprit vessel before PPCI in STEMI patients, most likely due to spontaneous reperfusion, resulted in enhanced levels of succinate in peripheral blood as compared with patients with TIMI flow ≤ 1. These data suggests that the concentration of this metabolite achieved in the peripheral blood of STEMI patients undergoing successful reperfusion increases over time, and that a delay before blood sampling might be needed to attain higher levels of this metabolite. Unfortunately, however, plasma concentrations of succinate and other metabolites obtained early after PPCI from peripheral blood does not correlate with the degree of irreversible injury (i.e., infarct size) or the size of the area at risk in these patients, and cannot be considered a predictor of CMR variables.

Previous studies have shown that succinate accumulates in ischemic tissues, including the myocardium^1–4^. At the onset of reperfusion succinate is rapidly oxidized to fumarate by forward succinate dehydrogenase activity. Succinate oxidation, in turn, induces a massive reverse electron transfer from mitochondrial complex II to complex I, leading to ROS production, mitochondrial permeability transition pore opening and cell death^1,2^. In fact, prevention of succinate accumulation during ischemia or of its oxidation during reperfusion has been demonstrated to reduce myocardial infarct size in several animal models, including isolated mice hearts and in pigs submitted to transient coronary occlusion^1,2,12–14^.

Importantly, part of the succinate that accumulated during myocardial ischemia is released into the bloodstream following reperfusion^5–8^, in a process that is dependent on monocarboxylate transporter 1 (MCT1) activity^7^. Indeed, it has been quantified that more than half of total succinate accumulated in the ischemic myocardium is released into the circulation during initial reperfusion, while about one-third is oxidized^6^. In agreement with this, succinate and other citric acid cycle metabolites can be detected in the interstitium during initial reperfusion in isolated rat hearts^15^, and in the blood of pigs subjected to transient coronary occlusion^8^. Furthermore, several citric acid cycle intermediates, including succinate, were shown to be increased in plasma from 27 STEMI patients, 2 to 48 h after PPCI^16^. In this regard, our present study, using ^1^H-NMR spectroscopy analysis, shows that STEMI patients with TIMI flow ≥ 2 at the time of PPCI (and therefore having experienced spontaneous reperfusion), had increased levels of succinate as compared with patients with TIMI ≤ 1, which may indicate that peripheral succinate concentration increases over time after reperfusion. These data suggests that some delay after reperfusion, before blood sampling, might be required to attain higher plasma metabolite concentrations in peripheral blood. However, the potential correlation between succinate concentration in peripheral plasma at longer time intervals after PPCI and the size of myocardial infarction or area at risk deserves further investigation. On the other hand, and in contrast with previous studies, other authors found a decrease in succinate and other citric acid cycle intermediates 1 h after symptom onset in the same type of patients^17^. Reasons for these discrepancies are unclear.

Kohlhauer and coworkers found increased concentration of succinate in the blood of STEMI patients obtained from a peripheral vein or the coronary sinus immediately after stent implantation^5^. A similar increase in succinate levels during initial reperfusion was described by our group in the blood of the great cardiac vein in a pig model of transient coronary occlusion^8^. However, whereas in the first study succinate plasma concentration at the coronary sinus correlated with edema volume, a surrogate of acute ischemic injury, but not with irreversible myocardial injury (i.e., myocardial infarct size at 6 months measured by CMR or troponins quantified during the first 48 h)^5^, in our previous study succinate concentration at the great cardiac vein correlated with infarct size and was reduced by protective maneuvers^8^. In contrast with these previous observations, our present analysis shows that succinate concentration in peripheral blood, obtained early after PPCI, does not correlate with the degree of irreversible injury (i.e., infarct size) or the size of the area at risk in STEMI patients undergoing PPCI, limiting the applicability of this metabolite as a prognostic biomarker in STEMI patients.

A possible explanation for the lack of correlation in our present study is that we measured metabolite concentrations in blood from a peripheral origin, as we tried to mimic the clinical situation in which a peripheral via is available in most, if not all, STEMI patients. In contrast, previous studies measured succinate concentrations at the coronary sinus^5,8^, where they may represent a better picture of what is happening into the area at risk. Indeed, succinate was found to be the only metabolite significantly increased in coronary sinus blood compared with peripheral venous blood in STEMI patients^5^. Whether its concentration at this location correlates with the area at risk (acute ischemic injury)^5^ or final infarct size^8^ deserves further investigation.

Lack of correlations in our present study might be also explained by methodological differences, as compared with previous ones. Whereas here CMR data were obtained 3–7 days after PPCI^9^, in the study by Kohlhauer and coworkers edema volume was quantified by T2-weighted CMR 2 days after PPCI^5^. However, T2-weighted edema may not constitute an accurate surrogate for the area at risk^18^, particularly when it is determined outside a time window ranging between 4 and 7 days post myocardial infarction^19^. Similarly, in our previous study in pigs, infarct size was measured by triphenyltetrazolium staining soon after coronary occlusion^8^, an experimental methodology very different to that used in the clinical context.

Succinate concentrations in plasma seem to have a great variability between studies. In the work by Sadagopan, serum succinate concentrations, as measured by liquid chromatography tandem mass spectroscopy (HPLC) in samples from hypertensive or diabetic patients, ranged between 1 and 8 µmol/L^20^, values similar to those found by Kohlhauer in patients with angina or acute myocardial infarction^5^. However, others have found, using the same technique, values around 1–3 mmol/L in patients with acute myocardial infarction or coronary artery disease, and undetectable levels in healthy controls^21^. No clear explanation is currently available for these discrepancies, apart from methodological errors. But even within the same work, huge variations have been described. D’Alessandro et al., also using HPLC, found mean values or 10.1 ± 22.7 µmol/L in a control population with traumatic injuries that was increased to 96.1 ± 144.2 µmol/L in deceased patients, but values ranged from below 5 to more than 200 µmol/L (40x)^22^. Similarly, patients with aortic diseases had a median of 35.15 µmol/L, significantly higher than healthy controls (15.30 µmol/L), but again values ranged from about 10 to near 200 µmol/L (20x)^23^, whereas Osuna-Prieto and coworkers showed that succinate plasma levels ranged from 11 to 130 µmol/L in young adults (11x)^11^. In our study we measured succinate concentrations by ^1^H-NMR spectroscopy in plasma, and our values were in the range of some of those previous studies (about 69 ± 7 µmol/L in TIMI 1 and 141 ± 58 in TIMI 2 patients), and near to those we found in plasma from a pig model of transient coronary occlusion (from 9.1 ± 0.9 to 27.8 ± 3.9 µmol/L) in blood from the great cardiac vein^8^, but also shows a high variability (from 40 to 423 µmol/L, 10x). Reasons for this high variability are unknown but may be due, in part, to the different conditions within each individual patient, to the analytical technique (NMR vs. HPLC), to the extraction method, or to the use of plasma vs. serum.

## Conclusions

In conclusion, the present data suggest that spontaneous reperfusion enhances succinate levels in peripheral blood from STEMI patients, but do not support the utility of succinate and other citric acid intermediates assessed early during PPCI in plasma from that origin as prognostic biomarkers in these patients.

## Data Availability

The datasets used and/or analysed during the current study are available from the corresponding authors on reasonable request.

## Abbreviations

BMI: Body mass index
BSA: Body surface area
CMR: Cardiac magnetic resonance
LCX: Left circumflex coronary artery
LDA: Left descending coronary artery
LV: Left ventricular
LVEF: Left ventricular ejection fraction
MCT1: Monocarboxylate transporter 1
MVO: Relative microvascular obstruction
OAD: Oral antidiabetic drugs
PPCI: Primary percutaneous coronary intervention
RCA: Right coronary artery
STEMI: ST-segment elevation myocardial infarction
